# Pharmacokinetic Analysis of Peptide-Modified Nanoparticles with Engineered Physicochemical Properties in a Mouse Model of Traumatic Brain Injury

**DOI:** 10.1208/s12248-021-00626-5

**Published:** 2021-08-16

**Authors:** Lauren E. Waggoner, Marianne I. Madias, Alan A. Hurtado, Ester J. Kwon

**Affiliations:** 1 Department of Nanoengineering, University of California San Diego, La Jolla , CA USA; 2 Department of Bioengineering, University of California San Diego, La Jolla , USA CA

**Keywords:** nanoparticles, peptides, pharmacokinetics, surface engineering, traumatic brain injury

## Abstract

Peptides are used to control the pharmacokinetic profiles of nanoparticles due to their ability to influence tissue accumulation and cellular interactions. However, beyond the study of specific peptides, there is a lack of understanding of how peptide physicochemical properties affect nanoparticle pharmacokinetics, particularly in the context of traumatic brain injury (TBI). We engineered nanoparticle surfaces with peptides that possess a range of physicochemical properties and evaluated their distribution after two routes of administration: direct injection into a healthy mouse brain and systemic delivery in a mouse model of TBI. In both administration routes, we found that peptide-modified nanoparticle pharmacokinetics were influenced by the charge characteristics of the peptide. When peptide-modified nanoparticles are delivered directly into the brain, nanoparticles modified with positively charged peptides displayed restricted distribution from the injection site compared to nanoparticles modified with neutral, zwitterionic, or negatively charged peptides. After intravenous administration in a TBI mouse model, positively charged peptide-modified nanoparticles accumulated more in off-target organs, including the heart, lung, and kidneys, than zwitterionic, neutral, or negatively charged peptide-modified nanoparticles. The increase in off-target organ accumulation of positively charged peptide-modified nanoparticles was concomitant with a relative decrease in accumulation in the injured brain compared to zwitterionic, neutral, or negatively charged peptide-modified nanoparticles. Understanding how nanoparticle pharmacokinetics are influenced by the physicochemical properties of peptides presented on the nanoparticle surface is relevant to the development of nanoparticle-based TBI therapeutics and broadly applicable to nanotherapeutic design, including synthetic nanoparticles and viruses.

**Graphical abstract:**

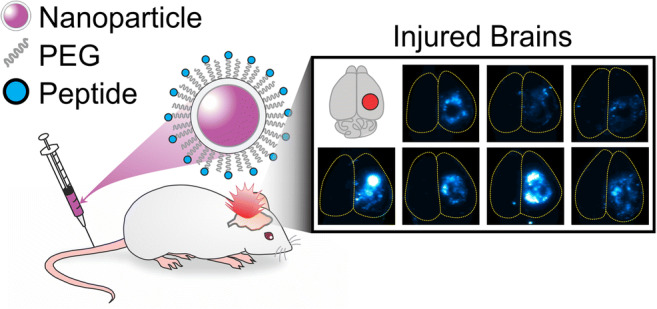

**Supplementary Information:**

The online version contains supplementary material available at 10.1208/s12248-021-00626-5.

## INTRODUCTION

Traumatic brain injury (TBI) affects more than 50 million people each year (1), yet there are currently no treatments for TBI that support long-term brain health (2,3). While the development of intravenously delivered therapeutics for the treatment of TBI is desirable for their ease of use, their clinical translation has been challenged by the poor pharmacokinetic profiles of TBI drugs, including limited bioavailability in the brain (4–6). Nanoparticle-based therapeutic systems are an attractive strategy for the delivery of drugs because as platform technologies, they have the potential to display pharmacokinetic profiles independent of their drug cargos. This independence is achieved through sequestering drug cargo in the core of the nanoparticle while controlling surface properties. Peptides are a promising class of molecules used to control nanoparticle surface properties and influence nanoparticle interactions with cells and tissues due to their biological activity and relatively small molecular size. Recent improvements in their good manufacturing practice (GMP) manufacture and chemistry to achieve long-term stability have made them tractable candidates for clinical translation (7,8).

In the context of TBI, peptide-mediated active targeting has been used to increase tissue- and cell type–specific accumulation and retention. A clinical hallmark of TBI is damage to the vasculature, allowing for nanoparticle access to the injured brain tissue through passive accumulation across the dysregulated blood-brain barrier (BBB) (9–11). Bharadwaj *et al*. investigated the size-dependent passive accumulation of PEG-modified polystyrene nanoparticles 20, 40, 100, and 500 nm in diameter after systemic administration in a controlled cortical impact (CCI) model and observed a significant decrease in nanoparticle accumulation when diameters were greater than 100 nm (12). Furthermore, nanoparticles can also be actively targeted to specific cell types or structures in the brain. For example, modification of nanoparticles with the rabies virus–derived peptide RVG (13,14) leads to neuronal tropism, as has been demonstrated for siRNA nanocomplexes and porous silicon nanoparticles delivered in mouse models of TBI (9,15,16). Nanoparticle platforms engineered with CAQK, a targeting peptide that binds to upregulated extracellular matrix components in the injured brain, improve delivery efficacy of siRNA and neuroprotective drug cargos to the site of injury after systemic administration (10,17). While the pharmacokinetics of targeted nanomaterials are often compared with control materials made with biologically inert, scrambled peptide sequences that share the same amino acid residues, and thus physicochemical properties (10,13), beyond the study of these pairs or small groups of peptides, there is a gap in understanding how the physicochemical properties of peptides influence nanoparticle pharmacokinetics and accumulation in the injured brain after TBI.

Modifications of the engineered nanoparticle surface with polymers, proteins, and targeting moieties can impart different physicochemical properties onto the nanoparticle, such as charge and hydrophobicity, which in turn changes pharmacokinetics such as biodistribution and cell-specific interactions (18). Recent efforts have been made to understand how the physicochemical properties of nanoparticles dictate biological interactions in the body, including the brain. In an evaluation of how engineered polymer surface properties changed nanoparticle tropism in brain cancer, Song *et al*. observed that nanoparticle surfaces with bio-adhesive aldehydes associated more readily with tumor cells and activated glial cells than nanoparticle surfaces with hydroxyl groups, indicating that nanoparticle surface chemistries influence their cellular interactions in the brain microenvironment (19). In a systematic study of the effects of physicochemical properties in nanotherapeutic vaccine development, Yamankurt *et al. *created a large library of ~1000 spherical nucleic acid (SNA) nanostructures and determined that lipid core and antigen compositions with differing charges changed the efficacy of antigen release from the core nanoparticle and subsequent immune activation, demonstrating that charged components of nanoparticle therapeutics can affect their interactions with complex biological systems (20). Biodistribution and passive tumor accumulation of micelles modified with anionic aspartic acid or cationic lysine residues mediated by the enhanced permeation and retention (EPR) effect were affected by nanoparticle charge in a mouse model of ovarian cancer (21). Passive nanoparticle accumulation into the brain after TBI via the dysregulated BBB post-injury has been compared to the EPR effect in solid tumors (10,11,22,23), suggesting that the physicochemical properties of peptide-modified nanoparticles may also affect nanoparticle passive accumulation in the injured brain after TBI. To our knowledge, there has not yet been a systematic study of how the physicochemical properties of peptides displayed on nanoparticle surfaces affect the pharmacokinetics of nanoparticles in a mouse model of TBI.

In the presented work, we study how the physicochemical properties of peptide-modified nanoparticles contribute to their biodistribution *in vivo*. When nanoparticle surfaces were functionalized with PEG and reacted with peptides that display a range of physicochemical properties, we observed that nanoparticle surfaces adopted the physicochemical properties of the peptides. In order to evaluate the pharmacokinetics of these peptide-modified nanoparticles, the material was directly injected into the healthy brain via convection-enhanced delivery (CED) or injected intravenously in a mouse model of TBI. We observed that the biodistributions of peptide-modified nanoparticles were influenced by peptide charge in both tested models. Nanoparticles modified with basic peptides had restricted distributions in the brain after CED when compared with nanoparticles modified with acidic, zwitterionic, or neutral peptides. After systemic administration in a mouse model of TBI, nanoparticles modified with basic peptides had elevated off-target organ accumulation and short blood half-lives leading to a relative decrease in brain accumulation. Comparatively, nanoparticles modified with acidic, zwitterionic, or neutral peptides demonstrated increased blood residence and increases in relative accumulation in injured *vs*. uninjured brain tissue after systemic administration. Our results suggest that peptide physicochemical properties, such as charge and hydrophobicity, should be considered when engineering therapeutic nanoparticles with peptide-modified surfaces. Peptides are promising tools to impart biological function onto nanoparticle therapeutics (e.g., targeting ligands, antigens for vaccines, receptor agonists) and furthering our understanding of how their physicochemical properties contribute to their biological interactions can broadly inform the design of nanoparticle-based therapeutics for pathologies such as TBI.

## MATERIALS AND METHODS

### Nanoparticle Surface Engineering and Characterization

Aminated 100-nm red or magenta fluorescent polystyrene nanoparticles (Magsphere, Inc.) were reacted with an excess of 5-kDa NHS-PEG-maleimide:NHS-PEG-methoxy (Laysan Bio, Inc.) at molar ratios 0:1, 1:10, 1:4, 1:1, and 1:0 in PBS at ~80,000 total PEG per nanoparticle for 30 min. PEG-modified nanoparticles were immediately purified with a Zeba Spin Desalting Column™ (Thermo Scientific™) with a 40-kDa size cut-off and reacted with cysteine-containing peptides (LifeTein, LLC) for 2–3 h before being purified of excess peptide. FAM-labeled peptide was used for absolute quantification of peptide modification. Nanoparticles used in *in vivo* experiments were additionally reacted with a near-infrared reporter VivoTag-750® (VT-750®) (PerkinElmer) before PEG modification. Purified nanoparticles were stored at 4°C until use.

Hydrodynamic diameters and zeta potentials were measured with a Zetasizer Nano ZS (Malvern Panalytical) in phosphate-buffered saline (PBS) or after a 30-min incubation at 37°C in 10% exosome-free newborn calf serum (NCS) in PBS. Exosomes were removed using a 100-kDa MWCO centrifugal filter (Microcon). Zeta potential was measured using the diffusion barrier method (24). Nanoparticle and peptide concentrations were determined via absorbance/fluorescence compared to known nanoparticle and peptide standards using a Spark multimode microplate reader (Tecan Trading AG, Switzerland).

Surface charge was also evaluated with a Rose Bengal gel shift assay. Equi-volumes of 0.25 mg/mL Rose Bengal dye and 1 mg/mL nanoparticles were incubated in PBS at room temperature for 1 h. For serum conditions, nanoparticles were incubated in 10% NCS in PBS prior to the addition of dye. Samples were run on a 2.5% agarose gel to analyze free Rose Bengal dye that did not adsorb to the nanoparticle surface. Gels were imaged on a BioRad scanner, and densitometric analysis of the gels was done in ImageJ.

### Convection-Enhanced Delivery of Peptide-Modified Nanoparticles

All animal experiments were approved by the University of California, San Diego Institutional Animal Care and Use Committee (IACUC). Eight-week-old female C57BL/6J mice (Jackson Labs) were secured in a stereotaxic frame under 2.5% isoflurane anesthesia, and a 0.5-mm hole was drilled 0.5 mm rostral and 1.75 mm right of bregma. A 24-gauge needle was inserted through the hole at a depth of 3 mm and allowed to equilibrate for 30 s. Mice were randomly assigned to 8 groups (*n* = 3), and 0.25 mg of peptide-modified nanoparticles was injected in 5 μL of PBS at 0.5 μL/min and allowed to equilibrate for 30 s before removal of the needle. Brains were harvested after perfusion with fixative 6 h post-injection to allow time for nanoparticle transport and cellular association. Cellular accumulation of polymeric nanoparticles administered via CED has been previously shown to increase between 4 and 24 h (19).

### Immunohistochemistry and Fluorescence Imaging

Brains were equilibrated in 30% w/v sucrose overnight and frozen in OCT (Tissue-Tek). Ten-micrometer-thick frozen coronal sections were taken at the site of injection and 0.5 mm and 1 mm rostral from the needle tract. Sections were counterstained with Hoechst, and tiled images were acquired on a Nikon Eclipse Ti2 (Nikon Instruments Inc.). Nanoparticle fluorescence was thresholded to correct for background fluorescence with ImageJ and a map of the signal from the three replicates was overlaid and the total area quantified for each replicate.

### Blood Clearance and Biodistribution in a Mouse Controlled Cortical Impact Model

8-week-old female C57BL/6J mice (Jackson Labs) were secured in a stereotaxic frame under 2.5% isoflurane anesthesia, and a 5-mm-diameter craniotomy was performed 2.0 mm caudal and 2.0 mm right of bregma. Controlled cortical impact (CCI) was performed with a 2-mm-diameter stainless steel piston tip at 3 m/s to a depth of 2 mm using an ImpactOne (Leica Biosystems). Mice were randomly assigned to 8 groups (*n* = 5 for biodistribution studies, *n* = 3 for blood half-life studies), and 40 mg/kg of control or peptide-modified nanoparticles was delivered via a tail-vein injection 6 h after injury. Control animals were injured and received PBS. Blood was collected from the tail-vein at 0, 5, 10, 15, 30, and 60 min after injection in 10-μL heparinized tubes (Drummond™). Organs were collected after perfusion with PBS 1 h post-injection to study nanoparticle accumulation in organs after intravenous administration. Previous studies have established organ accumulation of nanoparticles 1 h after systemic administration in TBI models (12,25).

### Blood and Tissue Analysis

Tissues were homogenized at 150–250 mg tissue per mL of Laemmli buffer with 100 mM dithiothreitol (DTT) and 2 mM ethylenediaminetetraacetic acid (EDTA) with a Tissue-Tearor handheld homogenizer (BioSpec) and heated to 90°C for 10 min. Peptide-modified nanoparticle concentrations in tissue homogenate and blood samples were quantified based on fluorescence of VT-750® compared to known nanoparticle concentrations using a LI-COR Odyssey (LI-COR Biosciences). Whole tissues were scanned for surface fluorescence before being processed for tissue homogenization.

### Statistical Analysis

Statistical analysis was performed on GraphPad Prism 9.1.2 software. Biodistribution of nanoparticles in each individual organ group was analyzed by one-way ANOVA with Bonferroni post-test.

## RESULTS

### Synthesis of Peptide-Modified Nanoparticles

**Figure 1:**
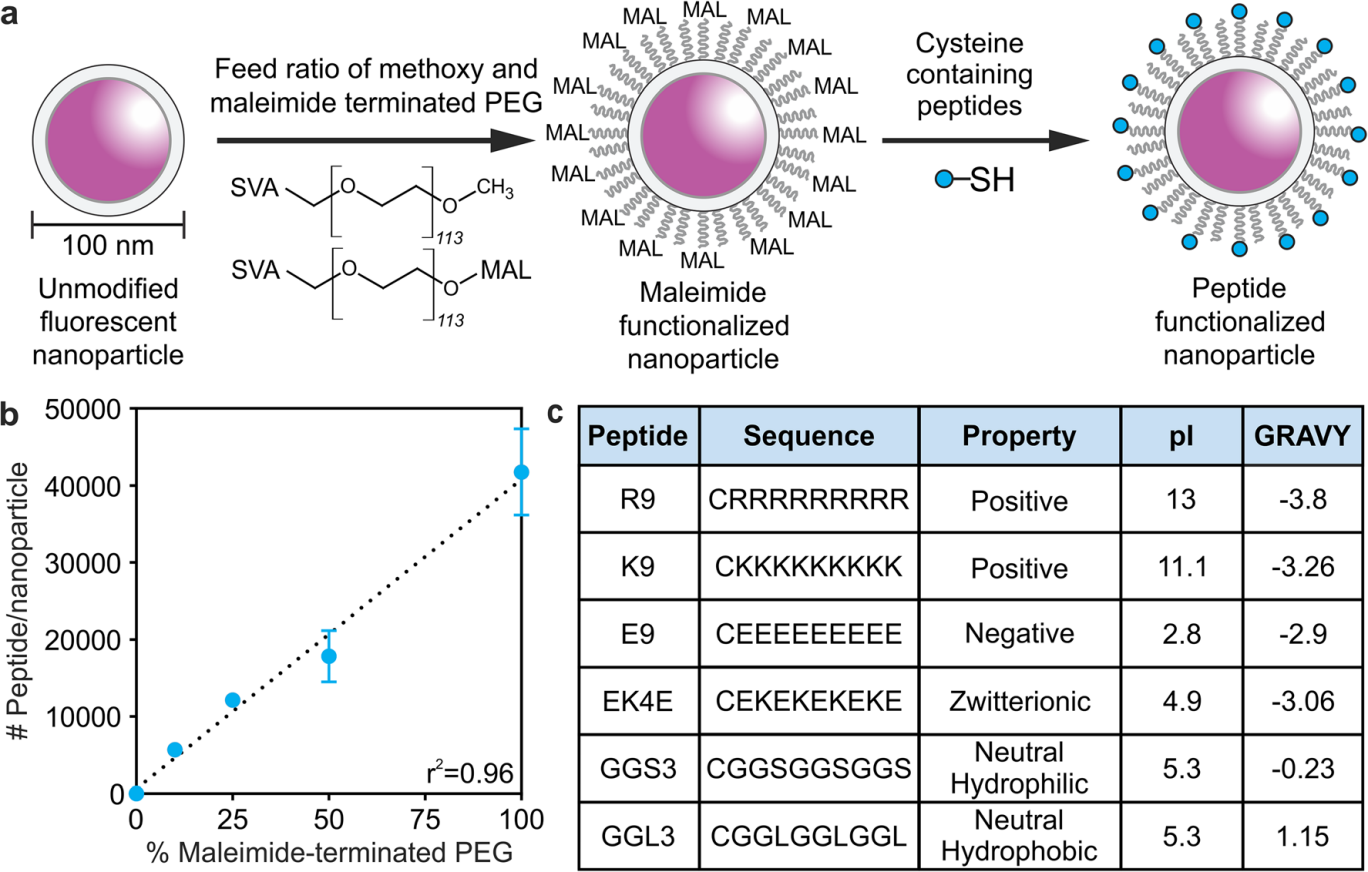
(**a**) Schematic of nanoparticle synthesis. Aminated nanoparticles were reacted with feed ratios of NHS-PEG and NHS-PEG-maleimide to form PEG-modified nanoparticle surfaces. Linear peptides with N-terminal cysteines were conjugated to the maleimide-terminated PEG. (**b**) Quantification of the number of peptides conjugated to nanoparticle surfaces with 0–100% maleimide-terminated PEG (*n* = 3, mean ± SD). (**c**) Peptides used in this study with their sequences, designed physicochemical properties, calculated isoelectric points, and GRAVY scores

Fluorescent polystyrene nanoparticles with aminated surfaces were used as a model nanoparticle for peptide modification based on ease of modification and fluorescence to allow for quantitative measurements of nanoparticle concentrations. Nanoparticles with 100-nm diameters were chosen based on previous studies that demonstrate nanoparticle accumulation in brain tissue after intravenous delivery in TBI animal models (11,12,26) and the similarity in size to existing FDA-approved therapeutics, such as Doxil® and ONPATTRO® (27,28). The aminated surfaces of the nanoparticle were fully reacted with an excess of 5-kDa NHS-PEG; PEG is a polymer used in many nanoparticle applications, including Doxil® and ONPATTRO® (28,29). The number of peptides per nanoparticle was quantified by synthesizing nanoparticles with various feed ratios of methoxy- to maleimide-terminated PEG followed by a reaction with a cysteine-bearing, fluorescein-labeled peptide to the distal end of the maleimide-terminated PEG (Figure 1a). Absolute numbers of peptides modified to the nanoparticle surface were quantified by measuring the absorbance of fluorescein from resulting nanoparticles compared to peptide standards (Figure 1b). We observed a linear correlation between the increasing proportion of maleimide-terminated PEG and the number of peptides (*r*^2^ = 0.96). We calculated that the resulting nanoparticles had a high PEG grafting density of 1.1 PEG/nm^2^ and ~18,000 peptides per nanoparticle when 50% of PEG chains were peptide-modified. In order to create peptide-modified nanoparticles that represent a range of physicochemical properties, the following peptide sequences were conjugated to 50% peptide-modified nanoparticles and used for subsequent studies: RRRRRRRRR (R9), KKKKKKKKK (K9), EEEEEEEEE (E9), EKEKEKEKE (EK4E), GGSGGSGGS (GGS3), and GGLGGLGGL (GGL3) (Figure 1c). Charge and hydrophobicity are physicochemical properties that influence pharmacokinetics and interactions with cell types and can be considered as universal design parameters when engineering therapeutic nanomaterials.

### Physicochemical Characterization of Peptide-Modified Nanoparticles

**Figure 2:**
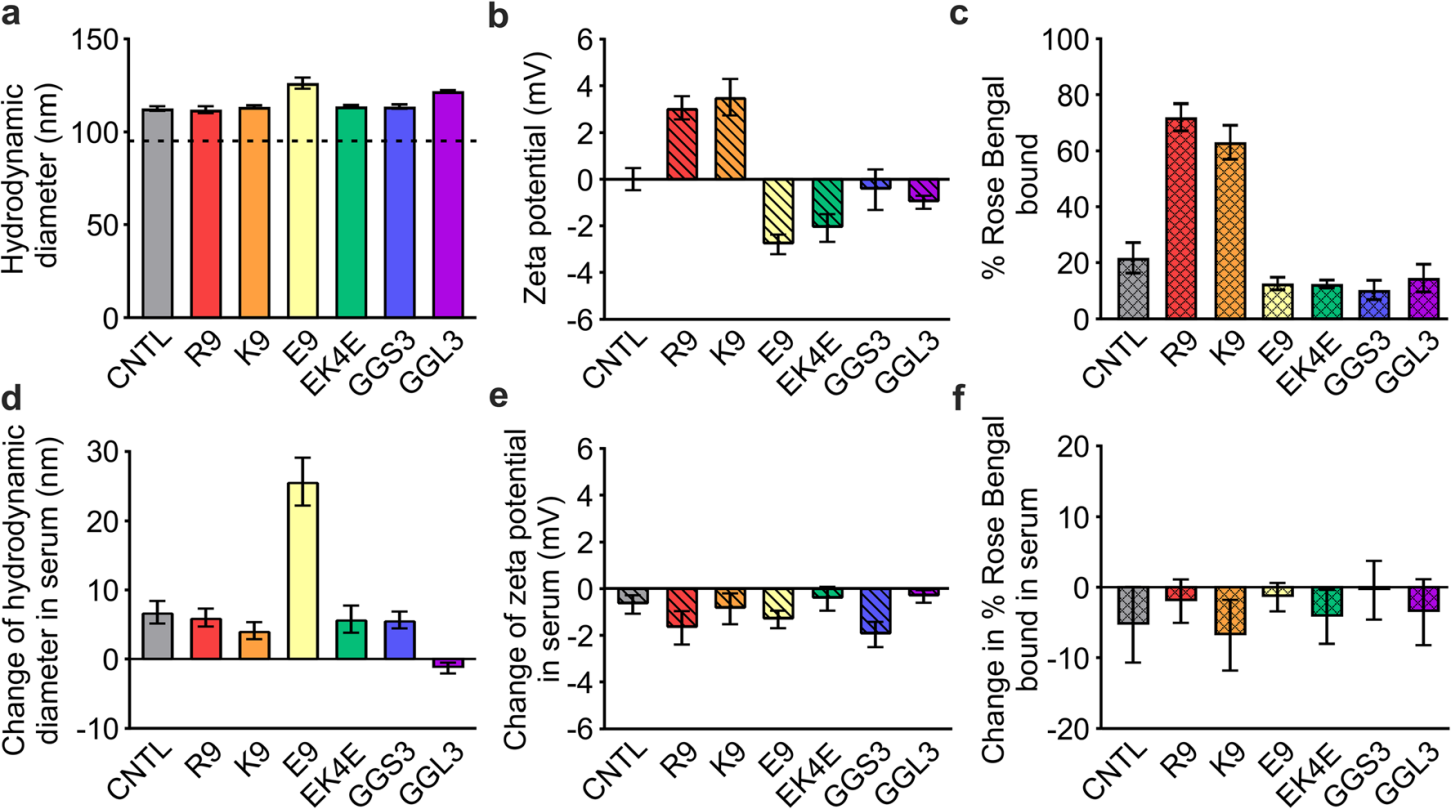
Hydrodynamic diameter (**a**), zeta potential (**b**), and percent Rose Bengal interaction (**c**) with peptide-modified nanoparticles measured in PBS. (**d**–**f**) Changes in hydrodynamic diameter, zeta potential, and Rose Bengal interaction after serum adsorption to nanoparticles (*n* = 3, mean ± SD)

The physicochemical properties of peptide-modified nanoparticles were characterized by dynamic light scattering (DLS) and Rose Bengal adsorption. The hydrodynamic diameter of unmodified polystyrene nanoparticles was 95 ± 1.5 nm and surface modification with PEG and peptide increased diameters ~20 nm (Figure 2a), consistent with the ~10 nm per molecule Flory radii of 5-kDa PEG in a brush conformation and linear peptide (30). Peptide conjugation imparted the expected characteristic charges of each peptide onto the surface of the nanoparticle; nanoparticles modified with basic peptides R9 and K9 displayed positive zeta potentials of 3.07 and 3.52 mV respectively, and nanoparticles modified with acidic peptide E9 displayed a negative zeta potential of −2.80 mV (Figure 2b). Nanoparticles modified with zwitterionic EK4E peptide also displayed a negative zeta potential of −2.09 mV, likely due to the additional terminal glutamic acid residue. Nanoparticles modified with neutral peptides GGS3 and GGL3 displayed near-neutral zeta potentials of −0.44 mV and −0.99 mV, respectively. Zeta potential measurements of peptide-modified nanoparticles compared to control nanoparticles modified with PEG and no peptide (0.01 mV) and unmodified aminated polystyrene nanoparticles (14.6 mV) indicate successful PEG modification and surface potentials that reflect the properties of the respective conjugated peptides. Rose Bengal adsorption assays have been previously used to characterize nanoparticle hydrophobicity and charge (31,32). We developed a Rose Bengal gel shift assay as an additional analysis of the peptide-modified nanoparticles. Nanoparticle interactions with Rose Bengal are largely driven by electrostatic interactions, due to the negative charge of Rose Bengal in experimental conditions (32). R9- and K9-modified nanoparticles formed interactions with 72.0% and 63.2% of the Rose Bengal dye, compared to the control nanoparticle, which interacted with 21.7% of the dye (Figure 2c), further confirming the basic character of R9- and K9-modified nanoparticles.

The adsorption of proteins onto nanoparticle surfaces or “protein coronas” in biological contexts has been an active area of research due to the impact of the protein corona on the biological activity of nanoparticles (33). Recent research has shown that the charge, hydrophobicity, size, and morphology of nanoparticles affect the composition of the protein corona (34–38). In order to understand how protein adsorption modulates the physicochemical properties of the peptide-modified nanoparticles, we repeated characterization after incubation of nanoparticles in 10% serum in PBS for 30 min at 37°C. Serum adsorption caused small changes in the hydrodynamic diameter of the nanoparticles (Figure 2d). After serum adsorption, the zeta potential of the peptide-modified nanoparticles consistently shifted to become slightly more negative by 0.33–1.95 mV (Figure 2e). Additionally, serum adsorption decreased nanoparticle interactions with Rose Bengal dye, consistent with our observed decreases in zeta potential measurements (Figure 2f).

### Peptide-Modified Nanoparticle Distribution in the Healthy Living Brain

**Figure 3:**
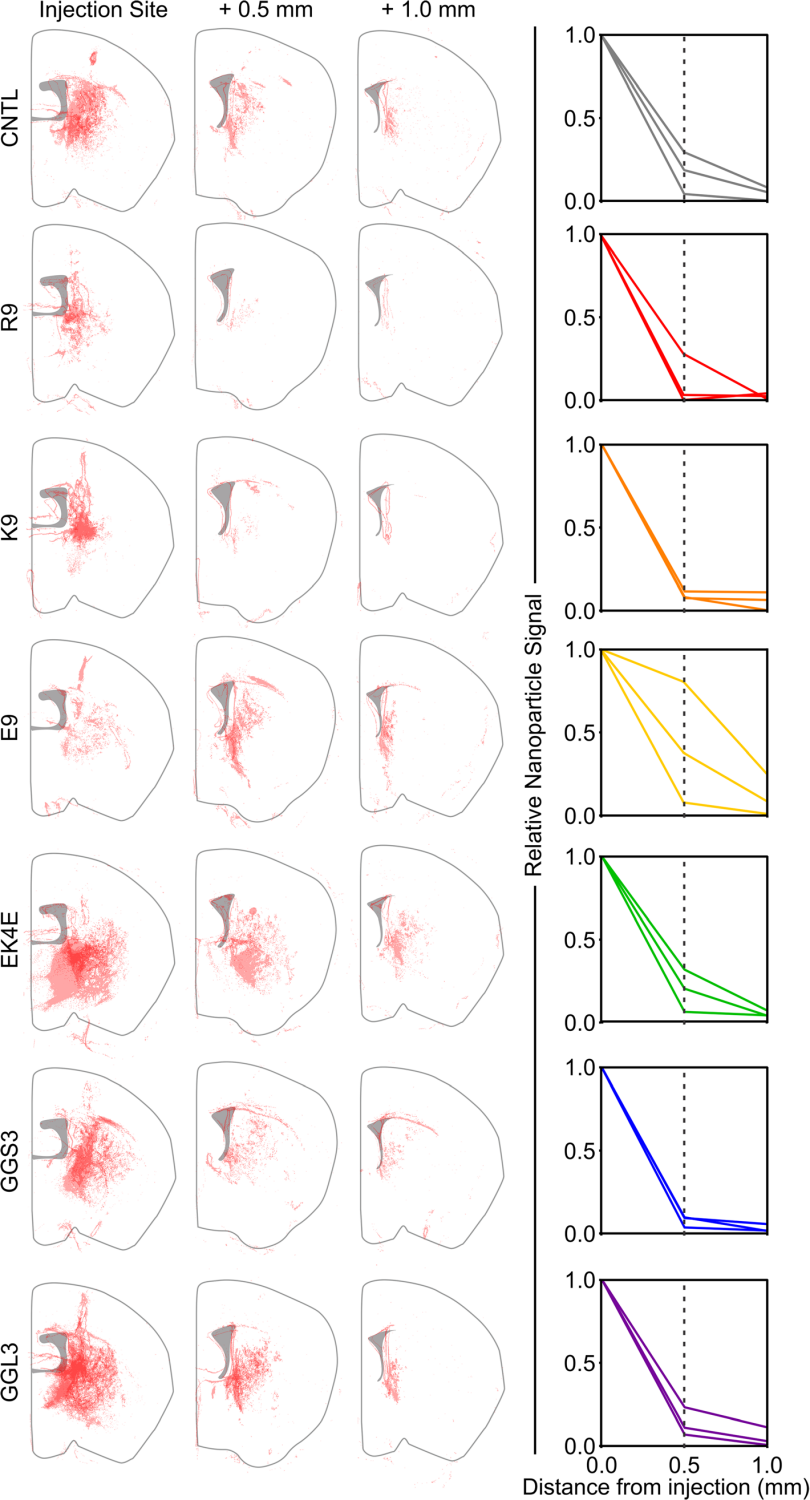
Distributions of peptide-modified nanoparticles after CED at 0, 0.5, and 1 mm away from the injection site (*n* = 3, each replicate depicted in red at 30% opacity). Distributions are overlaid on a schematic of a brain hemisphere. Right, relative areas of detected nanoparticle signal of peptide-modified nanoparticles as a function of distance from injection

We next sought to understand the distribution of peptide-modified nanoparticles in the complex microenvironment of the healthy living brain. Peptide-modified nanoparticles were administered via CED directly into the striatum of a healthy mouse brain, therefore bypassing the BBB. We studied the distribution of nanoparticles away from the injection site 6 h after injection to evaluate their relative mobility in the brain microenvironment. Coronal sections were taken at the injection site and 0.5 mm and 1 mm rostral from the injection site to ensure we were observing nanoparticles that had distributed away from the needle tract. We observed that R9- and K9-modified nanoparticles were not widely distributed in the analyzed brain sections (Figure 3, S1), indicating that nanoparticles modified with positively charged peptides had limited mobility from the injection site. In contrast, nanoparticles modified with neutral, negative, or zwitterionic peptides were distributed farther from the injection site after CED.

### Pharmacokinetics of Peptide-Modified Nanoparticles in a Mouse Model of TBI

**Figure 4:**
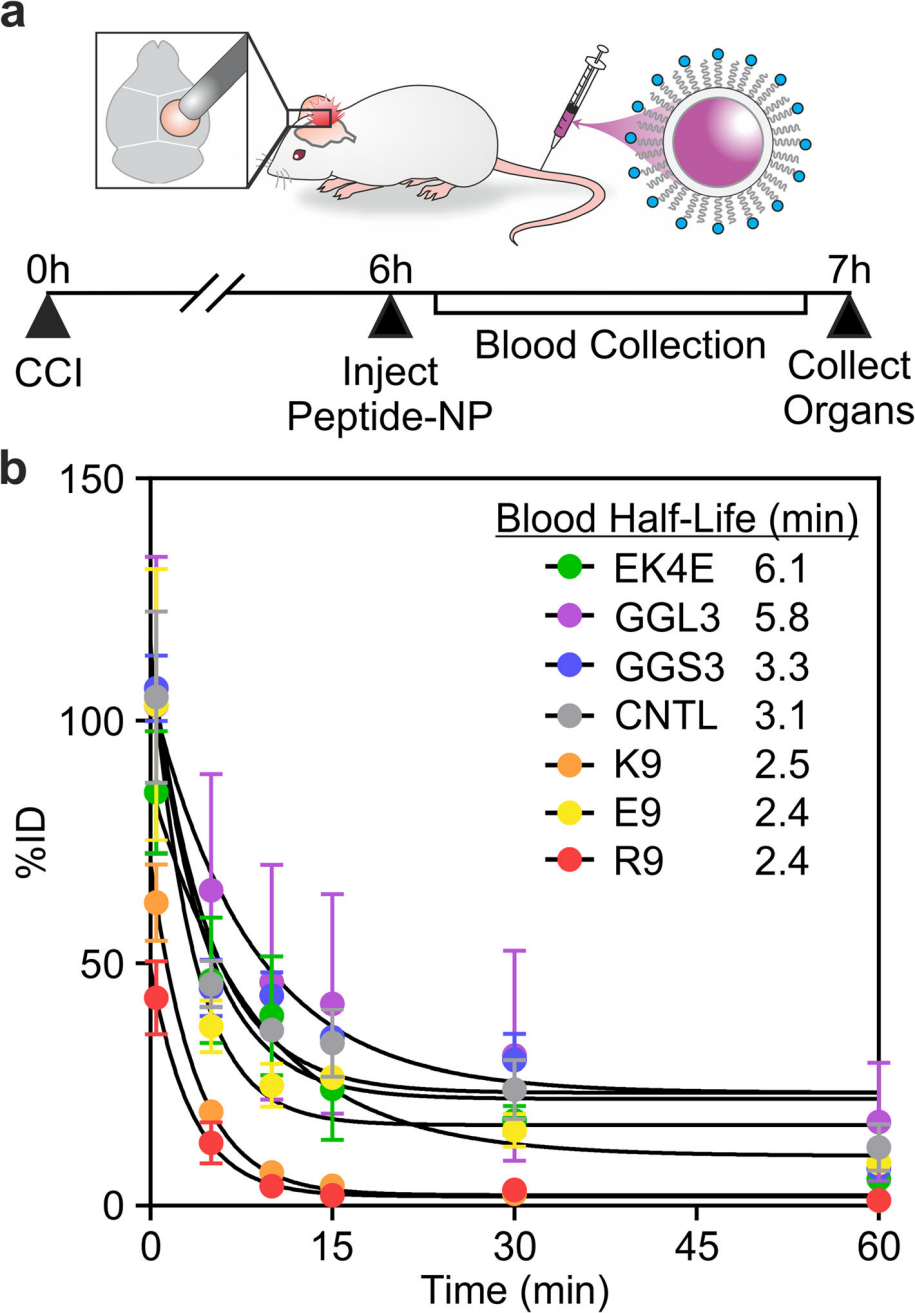
(**a**) Schematic and timeline of CCI, systemic peptide-modified nanoparticle administration, blood collection, and organ collection. (**b**) Percent injected dose of peptide-modified nanoparticles remaining in the blood at 0, 5, 10, 15, 30, and 60 min after administration with calculated blood half-lives (*n* = 3, mean ± SEM)

We next determined the effects of varying physicochemical properties of peptide-modified nanoparticles on nanoparticle pharmacokinetics after systemic delivery in a mouse model of TBI (Figure 4a). The right hemisphere of the brain was injured with a CCI, and mice were administered 40 mg/kg of nanoparticles or an equivalent volume of PBS via the tail-vein 6 h post-injury. In order to evaluate the blood half-life of the peptide-modified nanoparticles, blood samples were collected at 0, 5, 10, 15, 30, and 60 min after administration and nanoparticles were quantified based on their fluorescence signal (Figure 4b). The nanoparticles surface modified with the zwitterionic peptide, EK4E, had the longest blood half-life of 6.1 min. The neutral nanoparticles, modified with GGL3 or GGS3, and the control nanoparticle had blood half-lives of 5.8, 3.3, and 3.1 min, respectively. Nanoparticles with the largest absolute zeta potential values (K9-, R9-, and E9-modified nanoparticles) comparatively had the shortest blood half-lives between 2.4 and 2.5 min. K9- and R9-modified nanoparticle blood concentrations rapidly reached near-zero after 15 min, while the zwitterionic, neutral, and negatively charged nanoparticles maintained detectable concentrations in the blood up to the 60 min of measurement.

**Figure 5:**
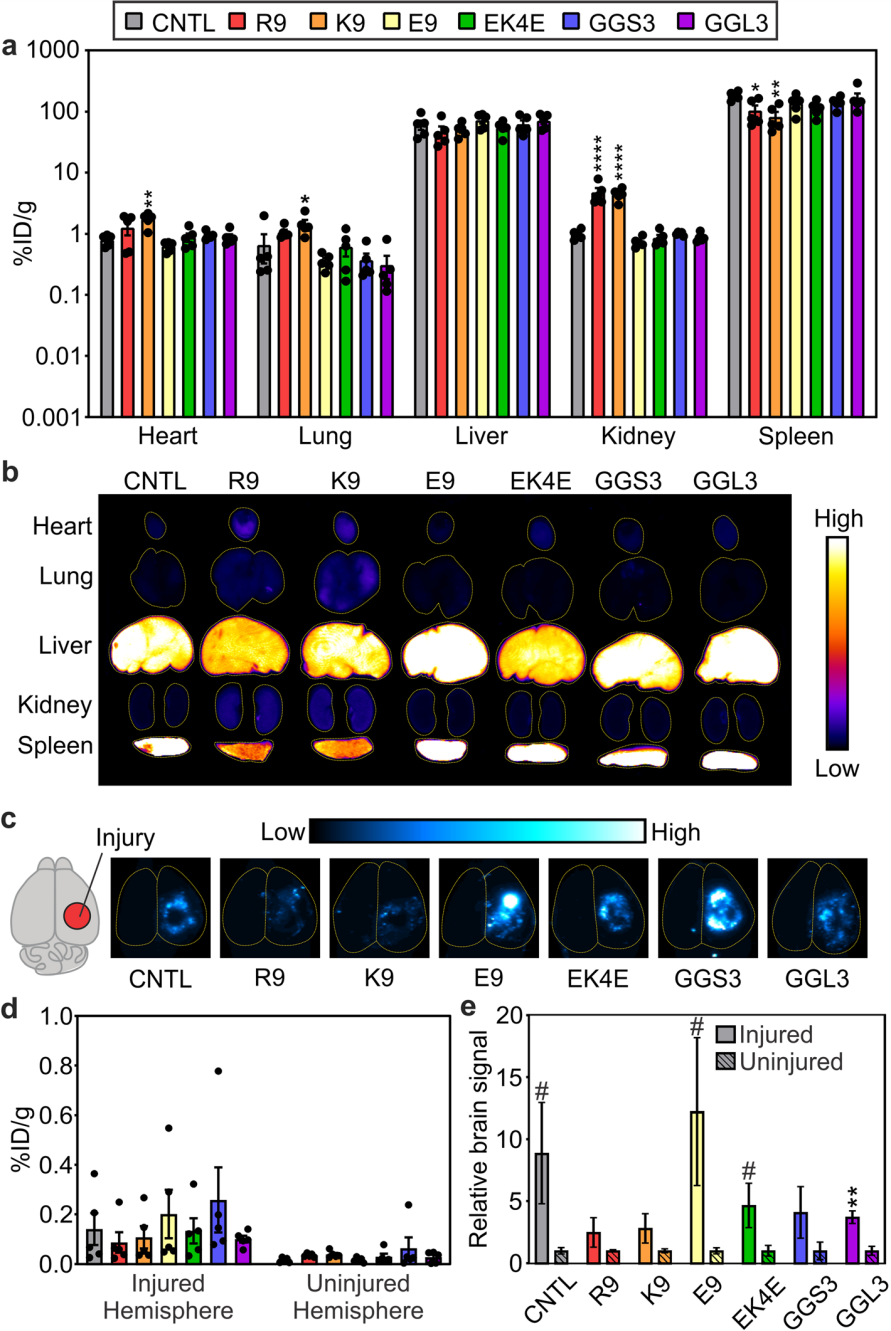
Accumulation of peptide-modified nanoparticles in dissociated organs 1 h after administration (**a**) and representative surface fluorescent images (*n* = 5, mean ± SEM; one-way ANOVA with Bonferroni post-test compared to control nanoparticles, *p < 0.05, **p < 0.01, ****p < 0.0001) (**b**). Representative surface fluorescent images (**c**) and accumulation of peptide-modified nanoparticles in dissociated brain tissue, separated by injured and contralateral hemispheres, 1 h after administration (**d**). Relative amounts of nanoparticle signal in the injured *vs*. contralateral uninjured hemisphere (*n* = 5, mean ± SEM; two-tailed *t* test between injured and uninjured groups, ^#^*p* < 0.1, **p < 0.01) (**e**)

Nanoparticle biodistribution was measured in homogenized tissue samples for quantification of bulk nanoparticle accumulation (Figure 5). Intact organs were also imaged prior to homogenization to provide spatial information of nanoparticle distribution on the surface of organs (Figure 5b, c, S2). The majority of observed signal from the accumulated nanoparticles in the brain is localized to the injured hemisphere (Figure 5c–e, S2). Neutral, zwitterionic, and negatively charged nanoparticles demonstrated more accumulation in the injured brain than positively charged nanoparticles. Additionally, R9- and K9-modified nanoparticles demonstrated increased accumulation in off-target organs such as the heart, lung, and kidneys compared to control, neutral, zwitterionic, or negatively charged nanoparticles (Figure 5a). Liver accumulation was similar for all nanoparticles.

## DISCUSSION

Nanoparticle interactions with biological environments have been engineered via surface peptide modification across multiple nanoparticle platforms, such as lipid nanoparticles (39–41), viruses (42), polymer nanoparticles (43,44), and porous silicon nanoparticles (15,16). While peptides have been studied individually in these contexts, there remain gaps in understanding how the physicochemical properties of the peptides affect nanoparticle pharmacokinetics. Furthermore, to our knowledge, this study is the first analysis of peptide-modified nanoparticle pharmacokinetics based on physicochemical properties in TBI models. We synthesized PEG-modified nanoparticles displaying peptides with characteristic charge and hydrophobicity (Figure 1a, c). We achieved a high density of PEG grafting on the surface of the nanoparticle (1.1 PEG/nm^2^); nanoparticles with PEG grafting densities ≥0.8 PEG/nm^2^ have been reported to avoid macrophage uptake *in vitro* and have increased blood half-lives *in vivo* (45). Peptide-modified nanoparticle physicochemical properties were confirmed to reflect the properties of the designed peptides when characterized by DLS and a Rose Bengal gel shift assay (Figure 2a, b, c). After pre-incubation with serum, peptide-modified nanoparticles had minimal increases in hydrodynamic diameter, a slight negative shift in zeta potential, and less interaction with Rose Bengal compared to their characterization in PBS (Figure 2d, e, f). PEG-modified nanoparticle surfaces have been shown to sterically hinder protein adsorption by repelling attachment with a hydrated shell that is formed in contact with biological fluids, leading to the formation of a minimal protein corona (46,47). The negative shift in zeta potential after serum adsorption we observed is supported by the majority of serum proteins being negatively charged, such as albumin, immunoglobulin, fibrinogen, and lipoproteins (48). Overall, peptide-modified nanoparticles displayed the expected physicochemical properties of their respective peptide, which were minimally affected by the adsorption of serum proteins.

Next, we studied the distribution profiles of peptide-modified nanoparticles in a healthy brain after CED to understand how the physicochemical properties of peptide surfaces affect their interactions with brain tissue. Nance *et al*. previously studied the diffusion of 40–200-nm polystyrene nanoparticles surface modified with a dense layer of PEG in the extracellular space of murine and human brain tissues (49). It was observed that nanoparticles with diameters up to 114 nm were able to diffuse through the brain, while diffusion was limited for particles 200 nm in diameter. Therefore, our objective was to understand how peptide physicochemical properties affected the transport of ~100-nm nanoparticles in the brain microenvironment. In coronal brain sections taken at the injection site and 0.5 mm and 1 mm rostral from the injection site, we observed that positively charged nanoparticles were less distributed through the brain tissue than neutral, zwitterionic, or negatively charged nanoparticles (Figure 3, S1). These observations are supported by previous findings that positive surface charge restricts liposome distribution in the brain microenvironment administered via CED compared to liposomes with negative and neutral surface charges (50). The limited mobility of positively charged nanoparticles away from the needle tract is likely due to their interactions with cells and extracellular matrix around the injection site, as positively charged nanoparticles can interact with negatively charged cell membranes (51–54). A similar phenomenon has also been described for the distribution of antibodies in a solid tumor; in the so-called binding-site barrier, high-affinity antibodies have limited mobility and penetration past the immediate cell layers adjacent to vasculature due to high-affinity binding (55). This also suggests that the reduced cellular association of neutral, zwitterionic, and negatively charged nanoparticles could contribute to increased nanoparticle distribution throughout the brain microenvironment, as their movement is less restricted by interactions with cells (56).

In particular, we studied how peptide physicochemical properties affect the pharmacokinetics of nanoparticles in an animal model of TBI. The CCI injury model is a well-characterized mouse model for TBI that results in tissue loss at the injury site and a transient increase in BBB permeability caused by vascular dysregulation following the injury (57–59). Although the extent of BBB dysregulation after injury is variable, significant nanoparticle accumulation within the brain has been previously reported for surface-modified and unmodified nanoparticles up to ~120 nm in hydrodynamic diameter when administered intravenously within 24 h post-injury (12,26). Peptide-modified nanoparticles were administered via the tail-vein 6 h after CCI injury, and blood samples were taken at time points over 1 h after injection to measure nanoparticle blood half-life (Figure 4a). Nanoparticles with zwitterionic peptide surfaces had the longest blood half-life, followed by nanoparticles with neutral peptide surfaces and finally nanoparticles with charged peptide surfaces (Figure 4b). Previous studies have established that zwitterionic nanoparticles repel serum protein adsorption, increasing their blood half-life compared to charged nanoparticles (60–62). Additionally, nanoparticles with greater absolute zeta potentials, E9-, K9-, and R9-modified nanoparticles, demonstrated shorter blood half-lives *in vivo* compared to more neutrally charged nanoparticles, likely due to their increased protein opsonization and subsequent macrophage uptake (51,63–65).

Organ biodistribution was established by measuring the fluorescence signal of nanoparticles in dissociated tissue, and the percent injected dose was calculated per gram of tissue (Figure 5a, d). Peptide-modified nanoparticle accumulation in the brain was more apparent in the injured hemisphere compared to the contralateral hemisphere (Figure 5c–e, S2), consistent with previous studies demonstrating that passive targeting of nanoparticles into the injured brain is localized to the site of injury (9,11,12). Fluorescent imaging of the brains also shows the localized accumulation of the peptide-modified nanoparticles proximal to the injury site, suggesting that accumulation is due to passive accumulation via the injured vasculature (Figure 5c, S2). Peptide modification of nanoparticles led to modest increases or reduced accumulation in the injured brain compared to the PEG-modified control nanoparticles without peptide (Figure 5d–e). Previous studies have demonstrated that passive accumulation of nanoparticles is dependent on reduced accumulation in off-target tissues (65–67), supporting the observation that cationic peptide-modified nanoparticles have less brain accumulation. However, the use of peptides for ligand targeting is commonly implemented in nanoparticle therapeutics to actively target cell types and biomolecules in the brain. Therefore, it is important to understand how the physicochemical properties of peptides may affect nanoparticle biodistribution and brain accumulation in models of TBI.

Positively charged peptide-modified nanoparticles have lower brain accumulation and elevated heart, lung, and kidney accumulation compared to neutral, zwitterionic, or negatively charged peptide-modified nanoparticles (Figure 5a, d). In previous biodistribution studies comparing charged nanoparticles, high absolute zeta potential and positive charge increased non-specific nanoparticle tissue accumulation (21,68,69). Accumulation of positively charged peptide-modified nanoparticles in off-target organs also likely contributed to their short blood half-lives and reduced passive accumulation in the injured brain. Similar pharmacokinetic profiles were described in a previous study of cell-penetrating peptides with basic character, where authors observed peptides localized to capillary-rich off-target organs, such as the liver, spleen, lung, and kidneys, and had short blood half-lives (70). Positively charged R9- and K9-modified nanoparticles have higher non-specific accumulation in cells and tissues, and previous studies have demonstrated that positively charged nanoparticles are more cytotoxic than neutral or negatively charged nanoparticles (71–73), indicating that nanoparticle toxicity should be carefully considered when designing nanoparticles with positively charged peptides. Although the extent of nanoparticle accumulation in injured brains exhibited a wide range due to the known variability of TBI animal models (74), nanoparticles modified with zwitterionic, neutral, or negatively charged peptides had modest increases in injured brain accumulation compared to nanoparticles modified with cationic peptides (Figure 5c–e). This effect may be due to the reduced accumulation of neutral, negative, and zwitterionic peptide-modified nanoparticles in off-target organs (Figure 5a) and improved blood retention when compared to R9- and K9-modified nanoparticles (Figure 4b). Previous research supports increased nanomaterial blood half-life with increased passive injury accumulation in TBI models due to the EPR-like effect in the injured tissue (11,26,75). Nanomaterials engineered to have long blood half-lives, such as PEG-modified materials, are also well-established nanomedicine platforms in cancer research due to their greater passive accumulation in solid tumors (65,66).

Interestingly, although the E9-modified nanoparticles have a shorter blood half-life comparable to the R9- and K9-modified nanoparticles, their brain and organ accumulation is similar to the accumulation of nanoparticles modified with zwitterionic and neutral peptides (Figure 4b, 5a, d). We observed a rapid decline in blood concentration of E9-modified nanoparticles within 10 min of circulation, followed by residual blood retention that was elevated compared to R9- and K9-modified nanoparticles. At the 60-min timepoint, E9-modified nanoparticles were comparatively 8-times more concentrated in the blood compared to basic peptide-modified nanoparticles, with 8.8% of the injected dose remaining in circulation. Interpretation of this data through a nonlinear clearance model, in which nanoparticles are sequestered from the blood by a limited number of available clearing sites, suggests that E9-modified nanoparticles may be saturating their binding sites in the reticuloendothelial system (RES) within 10 min, reducing nanoparticle elimination for the remaining circulation time. Similar effects have been observed in cancer research using RES blockades, in which decoy nanoparticles are injected prior to nanoparticle treatment to sequester plasma opsonins and saturate binding sites in off-target organs (76). RES blockades have successfully increased nanoparticle blood retention and tumor accumulation for nanoparticles using active and passive targeting techniques (76–78). Liver blockades have also been achieved by administering extremely large nanoparticle doses to saturate available binding sites while the nanoparticles are in circulation; Ouyang *et al*. delivered high doses of PEG-modified gold nanoparticles intravenously to elevate passive tumor accumulation and blood retention (79). Despite rapid initial depletion of E9-modified nanoparticles from the blood, they appear to have less binding site reservoirs in the heart, lung, and kidney compared to basic peptide-modified nanoparticles (Figure 5a), likely leading to increased passive accumulation observed in the injured brain (Figure 5c-e).

## CONCLUSION

Engineering nanotherapeutics is a promising approach for the development of TBI treatments with improved pharmacokinetics. Recent research has demonstrated that nanoparticles modified with targeting peptides, such as RVG and CAQK, improve accumulation in the injured brain after systemic delivery through a combination of active and passive targeting (9,10,15–17). In the current study, we demonstrate that peptide charge characteristics affect peptide-modified nanoparticle pharmacokinetics after direct application to the brain with CED and intravenous administration in a TBI animal model. Our observations suggest that nanoparticles surface modified with neutral, zwitterionic, or negatively charged peptides may have more selective delivery of therapeutic cargos in TBI, due to their reduced accumulation in off-target organs and more specific accumulation in the injured brain after systemic delivery and enhanced distribution in the brain after direct injection. Our work suggests peptide charge should be considered as a design parameter when engineering nanoparticle platforms with targeting peptides for systemic delivery of TBI therapeutics. A greater understanding of how peptide physicochemical properties on the surface of nanoparticles dictate their pharmacokinetic profiles is valuable for the engineering design of many types of therapeutic nanomaterials, including peptide-targeted synthetic materials and natural nanoparticles such as bacteriophage and viruses.

## Supplementary Information

ESM 1 (DOCX 1197 kb)
