# Supplementary material for: Pharmacokinetic Analysis of Peptide-Modified Nanoparticles with Engineered Physicochemical Properties in a Mouse Model of Traumatic Brain Injury
Source: AAPS J. 2021 Aug 16;23(5):100. doi: 10.1208/s12248-021-00626-5 (PMC8367032; doi:10.1208/s12248-021-00626-5)
Supplement: Supplementary file 1 — (DOCX 1197 kb) [file 12248_2021_626_MOESM1_ESM.docx]

Supplemental Information

Pharmacokinetic analysis of peptide-modified nanoparticles with engineered physicochemical properties in a mouse model of traumatic brain injury

Lauren E. Waggoner^1^, Marianne I. Madias^2^, Alan A. Hurtado^2^, Ester J. Kwon^2^*

Departments of ^1^Nanoengineering and ^2^Bioengineering, University of California San Diego, La Jolla, California 92093

*To whom correspondence should be addressed. Postal address; 9500 Gilman Drive, La Jolla, California, 92093; email: [ejkwon@ucsd.edu](mailto:ejkwon@ucsd.edu); phone: 1-(858) 246-0122


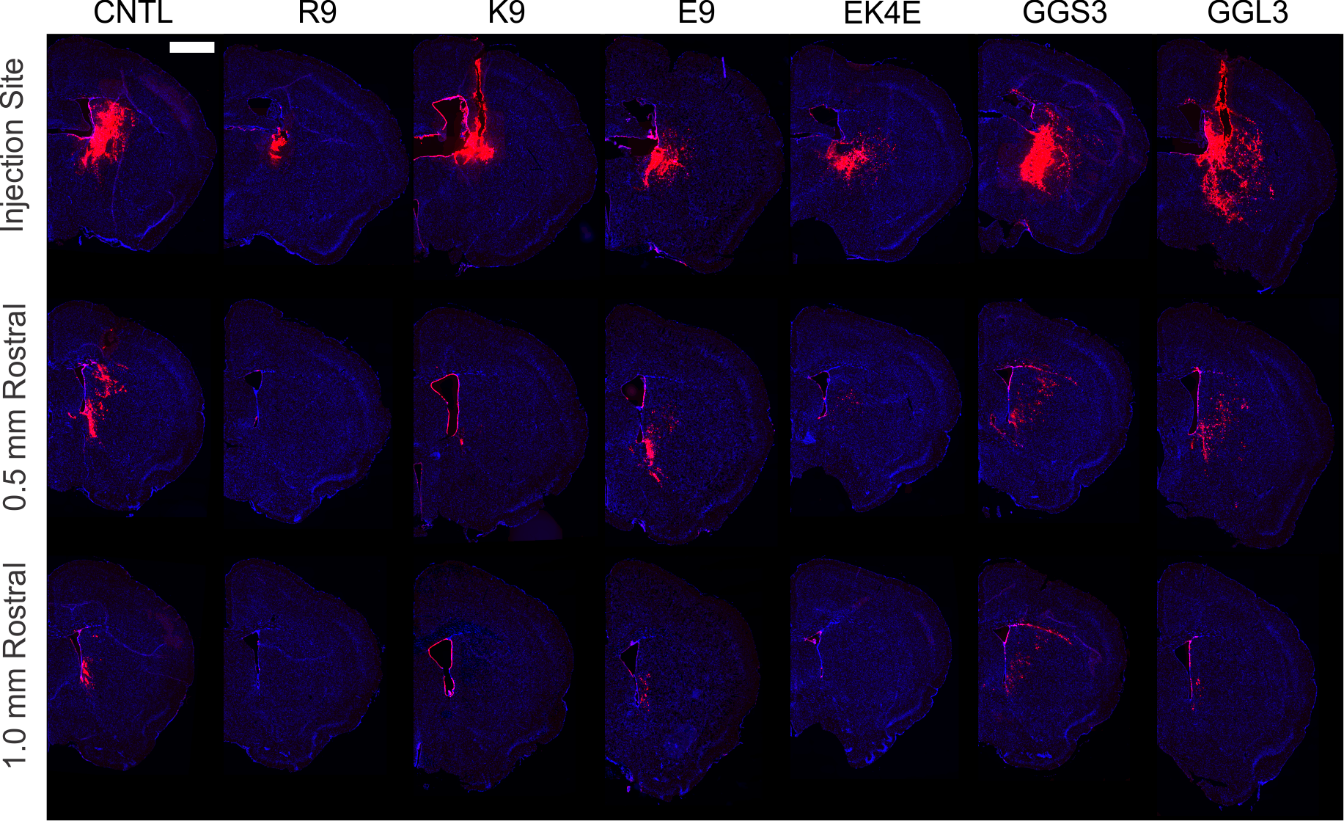


**Supplemental Figure 1.** Representative fluorescent images of brain sections taken at the injection site, 0.5 mm, and 1 mm rostral from the injection site 6 hours post-administration into a healthy mouse brain by convection enhanced delivery (CED) for each peptide-modified nanoparticle group (n=3). Injured brain hemisphere was imaged for nanoparticles (red) and Hoechst (blue; scale bar = 1 mm).


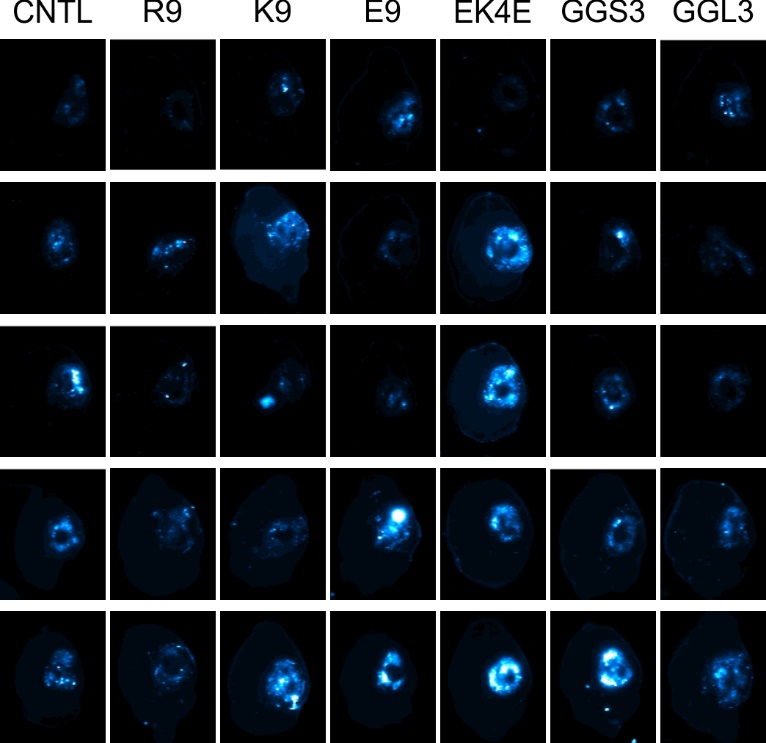


**Supplemental Figure 2.** Surface fluorescent imaging of intact brains, with injured hemisphere on the right side. Peptide-modified nanoparticles were administered intravenously 6 hours after controlled cortical impact (CCI) injury and brains collected 1 hour after administration.
